# South African family medicine research: Past, present and future

**DOI:** 10.4102/safp.v68i2.6300

**Published:** 2026-03-13

**Authors:** Robert J. Mash

**Affiliations:** 1 Division of Family Medicine and Primary Care, Faculty of Medicine and Health Sciences, Stellenbosch University, Cape Town, South Africa

**Keywords:** family medicine, research, clinical trials, research networks, primary care research, research capacity building

## Abstract

Over the past 45 years, the publication of original research in the *South African Family Practice* journal has steadily increased from humble beginnings in the 1980s to approximately 40–50 articles per year. This reflects the growth of the discipline and new speciality of family medicine as well as the shift in national policy towards primary health care as the foundation of the health system. Despite this growth, the profile of the research remains largely descriptive with surveys, audits and exploratory qualitative studies. The implication of this is that the discipline may not be addressing critical research questions on effectiveness, efficiency or identifying risk factors for common or important conditions. There is diversity of research questions and topics, although some areas remain relatively neglected, such as research on children, pregnant mothers, palliative care, rehabilitation, continuity and coordination.

**Contribution:**

As we look forward to the future, the discipline should be ready to analyse new electronic sources of ‘big data’, develop capacity for clinical trials and cost-effectiveness studies, launch more practice-based research networks, strengthen the whole research capacity-building pipeline and set priorities at a national level.

## Introduction

The *South African Family Practice* journal is 45 years old, and in this article, I look back at the research published in the journal and look forward to the work of the next generation of family medicine researchers.

## The past 45 years

**FIGURE 1:**
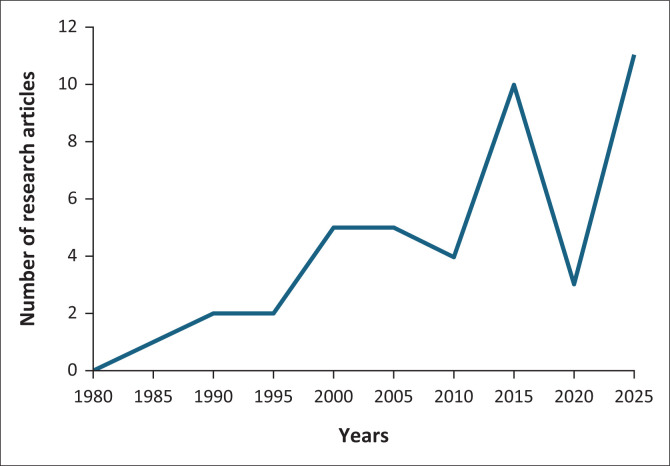
Number of original research articles published in January-February from 1980 to 2025.

Figure 1 shows the number of original research articles published every fifth year from 1980 to 2025, between January and February. In the 1980s, the journal did not really publish research. Many articles labelled as research were in fact continuing professional development articles. The few research articles published were eclectic in style and structure and would probably not pass peer review today. Much of the work was carried out by general practitioners describing their clinical practice. During this period, the discipline was largely led by private general practitioners with a few nascent departments of family medicine at the universities.

With the transition to a new South Africa in the 1990s and a new focus on primary health care and public sector services, there was little increase in the amount of research published. However, by the end of the decade, with more university departments of family medicine, emerging under- and postgraduate training, and doctoral programmes, there was a noticeable increase in research publications. By 2015, the output reached something of a ceiling with 10–11 publications in this 2-month period. In 2020, there was a hiatus as the journal changed publishers, and the coronavirus disease 2019 (COVID-19) pandemic may have also affected submissions. Over the whole of 2025, the journal published 53 original research articles.

Table 1 shows the types of study designs in the selected volumes from 1980 to 2025. Descriptive observational research in the form of surveys and audits dominates the field. Descriptive exploratory qualitative research also emerged strongly in the new millennium. Other study designs were identified but in very small numbers. One explanation could be that family medicine researchers are publishing their clinical trials and analytical observational studies elsewhere. However, our knowledge of the discipline and research field would suggest that this is not the case and that this is a true reflection of the methodological focus. Its implication is that the discipline is not addressing critical research questions on effectiveness, efficiency or identifying risk factors for common or important conditions.

**TABLE 1:** Types of study design between 10 January and February editions 1980–2025 (*N* = 43).

Study design	*n*	%
Descriptive observational such as surveys and/or audits	25	58
Descriptive exploratory qualitative	7	16
Analytical observational such as case-control	3	7
Phenomenology	2	5
Experimental such as randomised and non-randomised trials	2	5
Participatory action research	1	2
Review	1	2
Validation study	1	2
Mixed methods	1	2

## The present

A recent combined review of articles from 2020 to 2022 in the *South African Family Practice* journal and *African Journal of Primary Health Care and Family Medicine* analysed family practice research in Africa.^1^ In this article, I have extracted the data on the *South African Family Practice* journal to assess the profile of research published in this journal alone. During this period, the journal published 131 original research articles, between 41 and 47 per year. The majority of first authors were from South Africa (*n* = 116, 88.5%), with a smattering of authors from other African countries (e.g. Kenya, Malawi, Nigeria). As this is a national journal, representing the South African Academy of Family Physicians (SAAFP), this was expected. The mean number of authors per article was 3.4 (standard deviation [s.d.] 2.0), and the mean number of institutions per article 1.5 (s.d. 0.9).

Table 2 shows the characteristics of the articles. The authors were mainly from family medicine (55.7%), but public health, nursing, midwifery, educationalists and emergency medicine were in the top five contributors. In terms of the three components of primary health care, the majority were focused on integrated health services (*n* = 105, 80.2%), with a few looking at community empowerment (*n* = 16, 12.2%) and only one (0.8%) at multisectoral policy and action.

**TABLE 2:** Characteristics of research articles (*N* = 131).

Characteristics	*n*	%
**Authors’ disciplinary background**		
Family medicine	73	55.7
Public health	32	24.4
Nursing and midwifery	16	12.2
Health science education	13	9.9
Emergency medicine	8	6.1
Other	46	35.1
**Typology of research**		
Basic	0	0.0
Clinical	89	67.9
Service delivery	57	43.5
Health system	15	11.5
Education	19	14.5
**Focus on the life course**		
Pregnant mothers	7	5.3
Neonates	3	2.3
Infants	4	3.1
Children	6	4.6
Adolescents	13	9.9
Adults	66	50.4
Older adults	19	14.5
**Focus on the continuum of care**		
Health promotion	24	18.3
Disease prevention	26	19.8
Treatment	50	38.2
Rehabilitation	4	3.1
Palliative care	2	1.5

Most research focused on clinical topics (67.9%), and a substantial proportion looked at service delivery (43.5%) (Table 2). There was some attention given to health systems and educational research, but no publications on basic research. Basic research develops the tools needed for primary care research, for example, validating a tool to measure brief behaviour change counselling. The methodological profile was consistent with the previous 45 years. Most of the studies used descriptive or cross-sectional surveys (*n* = 85, 64.9%) or descriptive exploratory qualitative research (*n* = 22, 16.8%). Most studies looked at adults (64.9%), followed by adolescents (9.9%). There was little focus on maternal, neonatal and child health. In terms of the continuum of care, there was a solid focus on treatment issues (38.2%), as well as health promotion and disease prevention (38.1%), but very little work on physical rehabilitation or palliative care.

Table 3 breaks down the focus of the articles within the typology of research categories. Clinical research was well distributed across the burden of disease, with some emphasis on infectious diseases (e.g. human immunodeficiency virus [HIV], tuberculosis) and less emphasis on trauma and injury. Service delivery research mainly focused on the core functions of primary care (26.0%), and within this, the emphasis was on person-centredness and comprehensiveness. Given the methodological profile, it is not surprising that there was little published on effectiveness and efficiency. Within educational research, there was more focus on undergraduate educational issues, and little was published on the postgraduate programmes. Among the few publications on health systems, the focus was on the health workforce (*n* = 8, 6.1%).

**TABLE 3:** Specific focus areas within the research typology (*N* = 131).

Characteristics	*n*	%
**Clinical focus**		
Infectious diseases	26	19.8
Non-communicable diseases	18	13.7
Mental health	14	10.7
Maternal and women’s health	21	16.0
Trauma and injury	12	9.2
**Service delivery**		
Service design	6	4.6
Leadership and management	4	3.1
Community engagement	16	12.2
Quality improvement and patient safety	13	9.9
Resilience of facilities and services	6	4.6
Access and utilisation	7	5.3
Core primary care functions	34	26.0
Effectiveness	1	0.8
Efficiency	1	0.8
**Education**		
Undergraduate	10	7.6
Postgraduate	2	1.5
Continuing professional development	6	4.6

## The future

Family medicine research has become well established over the last 45 years in South Africa, but there is much room for further development. However, as the research field develops, there is no guarantee that researchers will publish in the *South African Family Practice* journal. As a successful national journal, it currently has a cite score of 2.0 and an impact factor of 1.4. It is the official journal of the SAAFP, the professional body for family physicians in South Africa, and as such is linked to this professional community. This assists with commitment to peer review and to engaging with this target audience. Much of the research is produced by Master’s and Doctoral students from the ten departments of family medicine in the country and therefore comes from relatively inexperienced researchers.

The analysis of the current research suggests some areas that may require attention in the future. Pregnant women, neonates, infants, children and adolescents are relatively neglected in the published research, and this could be because of the added burden of obtaining ethics approval and informed consent. Physical rehabilitation and palliative care from a family medicine perspective are also areas that could be developed. The new speciality of palliative care has grown out of family medicine, and this should stimulate more research in this area. It would be good to also see more basic research.

In terms of the specific focus areas, more research is needed on access, continuity and coordination of primary care, which would strengthen the evaluation of the core functions. When thinking more broadly about the implementation of primary health care as a ‘whole of society’ approach,^2^ researchers should think more about studying the empowerment of people and communities as well as multisectoral policy and action.

In terms of our methodological toolkit, the emerging field of implementation research is very important.^3^ Much of our research focuses on changes to the model of care or service delivery and how to successfully implement interventions that are known to be effective. Family medicine researchers seem at home with both quantitative and qualitative data, with mixed methods increasingly becoming the norm, particularly for doctoral studies. Innovative approaches to integrating and reporting the findings should be embraced by the journal.^4^

Moving forward, there are six strategic areas that family medicine researchers should consider:

- Analysis of ‘big data’.
- Developing capacity for clinical trials.
- Developing capacity for cost-effectiveness studies.
- Developing practice-based research networks.
- Strengthening the research pipeline.
- National priority setting and coordination.

### Analysis of ‘big data’

Electronic medical records and routinely collected electronic data are increasingly the norm, even in middle-income countries such as South Africa. There are plans to catalyse the amount of electronic data available through the implementation of national health insurance. The Western Cape has collated all electronically available data around a unique personal identifier to create an integrated database and ‘single patient viewer’ facility. Over the next decade, we should see a rapid increase in the volume of digital health and electronic data.

Groups such as the International Consortium of Primary Care Big Data (INTRePID) have been established to enable family physicians and primary care researchers to perform comparative research between countries.^5^ Much of the initial work has been on the impact of COVID-19, for example, on cervical cancer screening, mental health visits as well as sexual and reproductive health services. Departments within South Africa should prepare to embrace data science and develop the skills in observational study designs to analyse these emerging datasets.

### Developing capacity for clinical trials

With an increasing number of established family medicine researchers, it should be possible to secure international grants to address questions of efficacy and effectiveness. The World Health Organization (WHO) has recognised that many clinical trials fail to produce useful evidence and has published an action plan to improve the quality of clinical trials.^6^ At the same time, they want to see more relevant clinical trials in primary care and in low-income and middle-income countries. Too much of the evidence employed in primary care guidelines is generated in very different tertiary hospitals or specialised contexts. Over the next few years, we should see an increase in funds available to support this, as many of the global funders are part of this initiative.

As we prepare our capacity and resources to address these important research questions, we should also consider innovative new study designs that may be particularly appropriate to primary care. Designs such as adaptive platform trials allow the design to be adapted in real time and to test multiple interventions across the platform.^7^

### Developing capacity for cost-effective studies

South Africa is committed to funding effective primary health care, but resources are not limitless, and policymakers must make difficult decisions on what to prioritise. Indeed, in recent times, we have been living with austerity and a reduction in primary care funding for services as well as research. There is almost a complete absence of economic analysis in the published articles. Recommendations to change policy or introduce new services require economic evidence to assist policymakers in acting. Our research teams should include researchers with skills in economic evaluation and analysis (e.g. cost-effectiveness analysis), healthcare financing and systems, as well as health technology assessment.^8^

### Developing practice-based research networks

Most family physicians are in clinical practice and do not participate in research, although they were obligated to develop research skills as registrars. Only a few of these family physicians will develop a more academic career, complete a doctorate and become established researchers. Despite this, many clinicians identify important research questions on a regular basis from their practical experience, but have no means to address them. In the public sector, family physicians have a specific responsibility for clinical governance to improve the quality of care and patient safety. One strategy to assist with this is to perform applied research.

Practice-based research networks (PBRN) are increasingly recognised as an effective strategy to perform primary care research.^9^ One good example is the SUFPREN (Stellenbosch University Family Physician Research Network) in the Western Cape.^10^ Multiple family physicians in community health centres and district hospitals across all six districts participate in the network, now also including the University of Cape Town. Collectively, they brainstorm and prioritise the research questions they want to answer. They then collaborate with colleagues at the university departments of family medicine to assist with the study design, ethics and permissions. Family physicians then collect or provide the data. Everyone needs to provide only a small amount of data, which makes the study feasible for busy clinicians. The academic family physicians then analyse and draft a report. All participating family physicians help to shape the final article, become authors and assist with knowledge translation. Over the next decade, I hope to see most universities support PBRNs in their catchment areas. This will rapidly expand the number of family physicians involved in research and the number of relevant research articles.

### Strengthen the research pipeline

It may surprise many to learn that obtaining a doctoral degree is just the start of the journey to develop as a researcher. Our next-generation researchers typically start by completing a master’s and then a doctoral degree. Over the next decade, we should ensure that every department of family medicine in the country has two to three academics with doctoral degrees. This cohort of evolving researchers will improve our supervisory capacity and the quality of research at a master’s level. They also become the substrate from which our established researchers will emerge.

Post-doctoral scholarships are not financially or professionally feasible for family physicians or for most clinicians. We need strategies to enable clinician-scientists to combine clinical and research work. For those in more academic posts, they should continue to develop a body of research, ideally on the foundation of their doctoral studies. They should seek out regional and international collaborations and develop the ability to successfully compete for research grants. They will need the mentorship of established researchers and further training to become effective supervisors.

Each university should have a cadre of established researchers who mentor and support the next generation, keeping the pipeline flowing. At the same time, they may develop centres of excellence, research units or centres, and function as senior researchers who support a team. A full and healthy pipeline will generate more research and of a higher quality that can be published.

### National priority setting and coordination

The SAAFP has recently established a national research committee. Such a body should have oversight of the state of family medicine research in the country and promote some of the ideas listed above. Currently, the committee focus is on supporting doctoral-level research and building capacity, coordinating national research projects, and promoting PBRNs.

At the same time, it is important for our discipline and for primary care research to reach consensus on our research priorities. Such a conversation should of course involve multiple stakeholders, including government, the national research committee, funding agencies and the public. Over the next decade it would be helpful for the SAAFP to develop such a consensus that can guide future researchers.

## Conclusion

Over the last 45 years, the *South African Family Practice* journal has developed from publishing almost no research to a steady output of 40–50 original research articles a year. This mirrors the development of family medicine as an academic discipline in the country. Our current research output is strong, but overly focused on descriptive studies, and with several relatively neglected areas, such as palliative care, rehabilitation, children, adolescents, continuity, and coordination. We have a strong foundation to build on in the next few decades. Six key strategies to strengthen our research and publications in the journal include: skills in analysis of ‘big data’, developing capacity for clinical trials and cost-effectiveness studies, launching more PBRNs, strengthening the whole of our research pipeline and having a national body to coordinate and set priorities.
